# The polymorphism ZNF804A rs1344706 is differentially associated with negative symptoms domains in schizophrenia

**DOI:** 10.1192/j.eurpsy.2024.610

**Published:** 2024-08-27

**Authors:** T. Lezheiko, N. Kolesina, V. Golimbet

**Affiliations:** Clinical Genetics Laboratory, Mental Health Research Center, Moscow, Russian Federation

## Abstract

**Introduction:**

Negative symptoms (NS) are an important clinical characteristic of schizophrenia. In recent years, clinical research on NS has focused on their clinical heterogeneity. Based on two-factor analysis, it has been proposed to divide NS into abulia-apathy (AA) and expressive deficit (ED) domains. A number of studies have shown that these domains have different effects on the clinical features of schizophrenia, which suggests different pathophysiological mechanisms of their development. Neurobiological differences between AA and DE have been identified in neuroimaging and immunological studies but there is less research on the genetic background of NS.

**Objectives:**

To search for an association between the rs1344706 polymorphism of the zinc finger protein gene (ZNF804A) and the AA and ED subdomains. The rs1344706 polymorphism is one of the best-supported risk variants for schizophrenia. The risk genotype AA has been shown to be associated with clinical presentations of the disease.

**Methods:**

The study included 1116 (741 (66.3% women) patients with schizophrenia. The diagnosis was made according to ICD-10 criteria (item F20). The average age of the patients was 38.4 (13.6) years, age at disease onset was 26.1 (10.6) years. NS were assessed with the PANSS. The PANSS-derived AA domain consisted of Emotional withdrawal (PANSS item N2), Apathetic social withdrawal (N4), Active social avoidance (G16). The DE domain included Blunted affect (N1), Poor rapport (N3), Lack of spontaneity (N6), Mannerism and posturing (G5), Motor retardation (G7), Disturbance of volition (G13). Genotyping of the ZNF804A rs1344706 polymorphism was carried out using HRM-PCR. ANOVA with genotype and sex as independent variables, and age at the time of disease manifestation and its duration as covariates was used. Post hoc tests were performed using Bonferroni correction.

**Results:**

A significant effect of the rs1344706 polymorphism on the severity of symptoms in the AA domain was revealed (F=5.88, df=2, p=0.002). In carriers of the CC genotype, the severity of symptoms was significantly lower than in carriers of the AA genotype and the AC genotype (8.4(3.5), 9.4(7.4) and 8.8(3.5) points, respectively). This effect was independent of sex and was not mediated by age at onset or duration of disease. There was no effect of the rs1344706 polymorphism on the severity of symptoms in the ED domain.

**Conclusions:**

The association of the ZNF804A rs1344706 (A/C) polymorphism with NS of schizophrenia has not been reported so far though some studies have found the effect of this polymorphism on  PANSS positive symptoms and PANSS total score. The finding of the association with NS can be explained by the fact that the NS heterogeneity was taken into account in the present study.

**Disclosure of Interest:**

None Declared

